# Microwave cooking increases sulforaphane level in broccoli

**DOI:** 10.1002/fsn3.1493

**Published:** 2020-03-05

**Authors:** Yingjian Lu, Xinyi Pang, Tianbao Yang

**Affiliations:** ^1^ College of Food Science and Engineering Nanjing University of Finance and Economics Nanjing China; ^2^ Department of Nutrition and Food Science University of Maryland College Park MD USA; ^3^ Beltsville Agricultural Research Center Food Quality Laboratory U.S. Department of Agriculture Agricultural Research Service Beltsville MD USA

**Keywords:** broccoli, glucoraphanin, low‐temperature cooking, microwave cooking, sulforaphane

## Abstract

Sulforaphane (SFR), an anticarcinogenic compound, forms from the hydrolysis of glucoraphanin (GLR) in broccoli. Cooking methods have been shown to affect broccoli GLR and SFR levels, but little is known about the effect of lightly cooking processes on them. In this study, the effects of microwave and low‐temperature cooking on GLR and SFR contents in broccoli were investigated. Both microwaving and mild heating increased the GLR and SFR levels in broccoli compared to the raw samples (without any treatment). In particular, SFR level was significantly low under 40°C and dramatically increased from 40 to 60°C, but nothing was detected at 70°C. Compared with conventional heating, microwave heating increased the GLR and SFR yield by about 80% at 50 and 60°C. Microwave power level also influenced the SFR contents. At the same temperatures (50 and 60°C), high‐power microwave (950 W) with a short time produced over 40% more SFR than low‐power microwave treatment (475 W). Hence, mild heating by microwave could increase the GLR and SFR levels in broccoli, and high‐power microwave heating with temperature control at 60°C could retain higher bioavailability of these bioactive compounds in broccoli.

## Graphical abstract

Microwaving and mild heating increased glucoraphanin and sulforaphane level in broccoli compared to the no‐treatment control. The optimal temperature is around 60°C. The microwave intensity (950 W) is better than low intensity (475 W).

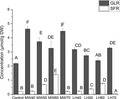

## 1 INTRODUCTION

**Figure 1:**
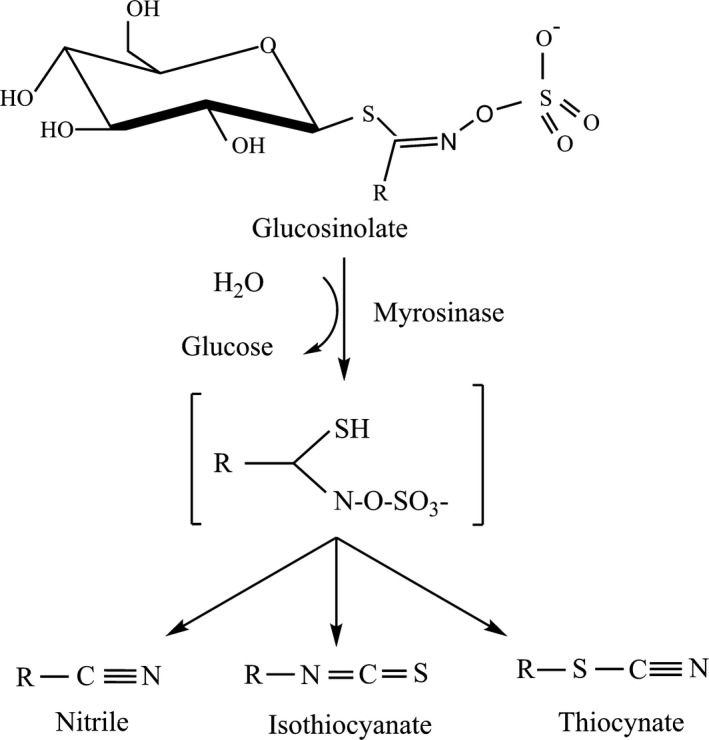
The general structure of glucosinolates and their hydrolysis products including nitrile, isothiocyanates, and thiocyanate by enzyme myrosinase

Broccoli contains a group of secondary metabolites called glucosinolates (GSL). The basic chemical structure of GSL is depicted in Figure 1. GSL consist a β‐D‐thioglucose group, a sulphonated oxime moiety, and a side chain derived from methionine, an aromatic or a branched amino acid (Moreno, Carvajal, López‐Berenguer, & García‐Viguera, 2006). In broccoli, the most abundant GSL is glucoraphanin (GLR, 80% of total GSL), which is an aliphatic GSL. Other GSLs include glucobrassicin (7.8%), 4‐methoxy‐glucobrassicin (3.8%), and 1‐methoxy‐glucobrasicin (3.8%), which are indolyl GSL (Cieślik, Leszczyńska, Filipiak‐Florkiewicz, Sikora, & Pisulewski, 2007). The anticarcinogenic effects brought by consuming brassica vegetables are not caused by GSLs, but due to their hydrolysis products, which are produced after GSLs being further processed by the enzyme myrosinase (MYR), including isothiocyanates (Figure 1). The major isothiocyanates found in broccoli are sulforaphane (SFR), which comes from the hydrolysis of glucoraphanin (4‐methylsulphinyl) by MYR, and are recognized as the most powerful cancer‐preventive agent.

Broccoli is mainly consumed and cooked by thermal treatment including boiling, steaming, and microwaving. These cooking processes could induce physical disruption of broccoli, leading to loss of cellular compartmentalization and mixing of GSL and MYR, which are separated from each other by vacuole membrane (Jones, Faragher, & Winkler, 2006). However, the GSL‐MYR system in broccoli could be modified by cooking processes leading to loss of phytochemical contents, as the result of enzyme action, leaching of water‐soluble GSL into cooking water, and thermal breakdown (Rungapamestry, Duncan, Fuller, & Ratcliffe, 2007). For instance, a reduction by 58%–77% of total GLRs was detected in broccoli after boiling for 30 min, with about 90% of the losses leaching into cooking water (Song & Thornalley, 2007). Over 90% loss of SFR was also detected in broccoli boiled for 2 and 5 min due to the inhibition of MYR (Jones, Frisina, Winkler, Imsic, & Tomkins, 2010). Because MYR is heat sensitive, intensive heat treatments such as boiling for more than 1 min and steaming for 4–5 min could inactivate the enzyme activity of MYR (Wang, Farnham, & Jeffery, 2012).

The microwaving has become a popular alternative way of cooking due to its convenience and high efficiency. In microwave heating, materials can absorb the energy directly and convert it into heat, resulting in faster heating rates compared to conventional heating (Vadivambal & Jayas, 2007). Previous studies reported controversial results about the effect of microwaving on the bioactive compounds in broccoli. It was reported that 60%‐74% of total GSL in broccoli was lost after microwaving for 5 min at high power (1,000 W) (Vallejo et al., 2002; Yuan, Sun, Yuan, & Wang, 2009). Also, the content of GLR within broccoli florets, regardless of difference between cultivars, is significantly reduced after microwave heating for 2 or 5 min at 1,000 W (Jones et al., 2010). However, Song and Thornalley (2007) found that microwave cooking at 900 W produced no significant loss of GSL in broccoli up to 3 min. The divergence may be due to different cooking conditions including heating time, microwave power, cut sample sizes, and physical structure.

Regarding the actual biologically active compound SFR, the microwave treatment at 600 W for 11 min caused a 38.9% reduction of SFR in broccoli (Galgano, Favati, Caruso, Pietrafesa, & Natella, 2007). According to Jones et al. (2010), microwaving at 1,100 W for 2 or 5 min could reduce the SFR concentration to the residual level because the internal temperature of microwaved broccoli could reach above the MYR denaturation temperature (70°C) less than 50 s. With shorter microwaving time, Wang et al. (2012) found that microwave heating (900 W) between 0.5 and 0.75 min increased production of SFR, followed by decrease from the maximum within 1 min. The effect of microwaving time on SFR production was also recently reported by Tab art, Pincemail, Kevers, Defraigne, and Dommes (2018) who found that SFR content in broccoli increased 4 times after 1 min of microwaving (950 W) and became undetectable after 3 min. In fact, the time–power combinations of microwave with same energy inputs could play an important role in the activity of MYR (Verkerk & Dekker, 2004). Thus, microwave settings could have great impact on the levels of GSL and SFR in broccoli.

Despite many studies on different cooking processes in terms of the GSL contents of broccoli, little research has evaluated the effect of mild thermal treatment achieved by common domestic cooking methods on the GLR and SFR contents. This study determines the effect of temperature and power level on GLR and SFR contents in broccoli microgreens during microwave treatment and compared these two bioactive compounds in florets and stems of broccoli.

## 2 MATERIALS AND METHODS

### 2.1 Chemicals

Formic acid and high‐performance liquid chromatography (HPLC)‐grade methanol and acetonitrile were purchased from VWR International, Inc. HPLC‐grade water was prepared from distilled water using a Milli‐Q system (Millipore Lab.). Glucoraphanin (GLR) potassium salt was purchased from Chromadex.

### 2.2 Plant materials and samples preparation

The mature broccolis were purchased from local grocery stores (Silver Spring). Broccolis were prepared by removing the inedible parts and then cut into homogeneous pieces. To obtain more homogeneous samples, each vegetable was prepared in batches of 200 g of the same purchased vegetables for each experiment.

### 2.3 Cooking treatments

Two of the most cooking methods used by modern population, different temperature and microwave cooking, were used. Cooking conditions were optimized by preliminary experiments carried out for each vegetable. Cooking process was obtained by monitoring the temperature strictly. Ten g of broccoli samples was submerged into 500 ml of water and then subjected to the following cooking treatments. The specific parameters were summarized in Table S1. Broccoli crowns were separated as florets only, stems only, and florets together with stems.

Low‐temperature cooking: Broccoli was added into water in a covered pot and heated to 40, 50, 60, and 70°C using low‐temperature cooking.

Microwave cooking: Broccoli was immersed into water and cooked in microwave oven at high power level (950 W) or low power level (475 W) and heated to 40, 50, 60, and 70°C.

After cooking, the samples were frozen in liquid nitrogen and freeze‐dried before storage at −20°C before analysis. In addition, two of these treated samples were not cooked and used as reference materials. For all parameters tested, we used two batches.

GLs in freeze‐dried vegetables was extracted following a previously reported procedure (Lu et al., 2018) with modification. Briefly, powdered samples (200 mg) were extracted with 5 ml of methanol − water (60:40, v/v) using sonication for 30 min at 70°C. After being cooled in an ice bath, the extracts were centrifuged at 10,000 × *g* for 15 min, and the supernatant was filtered through a 0.45 μm nylon filter (Waters Associates) for further ultra‐high‐performance liquid chromatography (UHPLC) and electrospray ionization (ESI)/ion‐trap mass spectrometry (ITMS) analysis.

### 2.4 UHPLC‐ESI/ITMS conditions

The UHPLC‐ESI/ITMS was performed according to a previously reported laboratory procedure (Sun et al., 2015). In brief, a LC/MS system consisting of an Agilent 1200 UHPLC system with a binary pump, a diode array detector (DAD), a vacuum degasser, a column oven, and an autosampler (Agilent Technologies) in combination with a LCQ Deca ion‐trap mass spectrometer (Thermo Fisher Scientific Inc.) was used for both LC and MS spectrometry.

The UHPLC conditions were as follows: A symmetry C18 column (2.1 mm × 150 mm, 3.5 µm) (Waters) was used with a column temperature set to 40°C. Mobile phase A consisted of 0.1% formic acid in H_2_O, and mobile phase B consisted of 0.1% formic acid in acetonitrile. The initial percent of B was 2%; this was changed linearly to 30% B in 20 min, increased linearly to 90% B in 25 min, back to 2% B in 26 min. The gradient was kept at 95% B for 5 min washing and returned to initial conditions for 5 min to re‐equilibrate the column for the next injection. The flow rate was 0.7 ml/min, and injection volume was 20 µl. The DAD wavelength was set to 229 nm since the highest absorbance for the reference peak was observed at this wavelength.

The MS in full scan mode (mass range 100–700) was also used to assist peak identification. The conditions for ESI/ITMS were as follows: sheath gas flowrate 80 (arbitrary units); aux and sweep gas at 15 (arbitrary units); spray voltage, 3.5 kV; heated capillary temperature, 250°C; capillary voltage, 4.0 V; and tube lens offset, 20 V. MS spectra were collected from 0 to 26 min, and the mass range was from 100 to 700 m/z. ESI in negative ion mode was used.

### 2.5 Qualitative and quantitative analysis of glucoraphanin and sulforaphane

GLR and SFR were determined using a UHPLC‐ESI/ITMS method. The GLR and SFR were identified by their MS spectra, UV‐visible spectra, and the order of elution previously described for similar acquisition conditions. The amounts of GLR and SFR were quantified using known concentrations of commercial standards by integration of the peak areas using Xcalibur 2.3 software (Thermo Fisher Scientific Inc., Waltham, MA, USA).

### 2.6 Statistical analysis

All the data are represented as average ± standard deviation (*n* = 3). Differences between means were determined by analysis of variance (ANOVA) with Tukey's HSD post hoc test (*p* < .05), using SPSS (SPSS for Windows, Version Rel. 10.0.5., 1999, SPSS Inc.). Correlation analyses were performed using a two‐tailed Pearson correlation test. Statistical significance was declared at *p* < .05.

## 3 RESULTS

### 3.1 Effect of cooking temperature on GLR and SFR levels in broccoli

**Figure 2:**
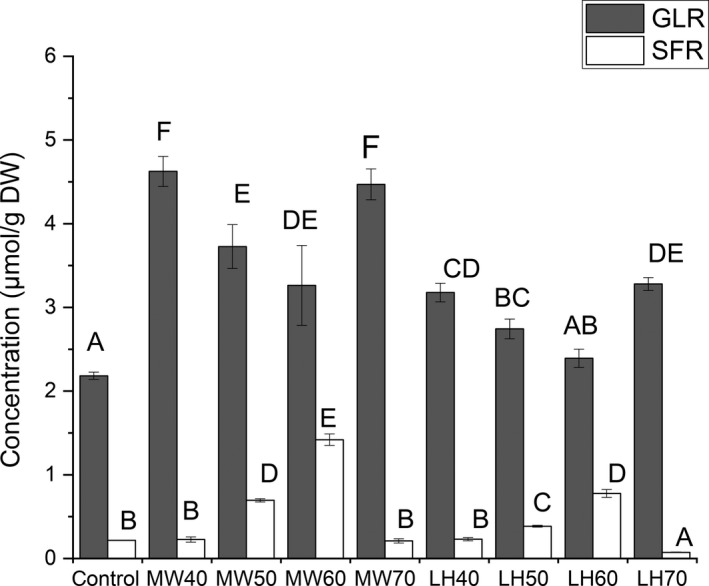
The glucoraphanin (GLR) and sulforaphane (SFR) contents (μmol/g DW) in florets of broccoli during microwaving (MW) and low‐temperature heating (LH) at 40, 50, 60, and 70°C. Data are reported as the mean ± *SD* (*n* = 3). Values with different letters are significantly (*p *< .05) different

Since florets are the major edible part of broccoli crown, the levels of GLR and SFR in broccoli florets cooked at different temperatures were determined (Figure 2). Results showed that the broccoli cooked using microwave at 40°C showed the highest GLR at 4.89 µmol/g DW. The broccoli sample without cooking as a control showed the least amount of GLR, indicating that microwave heating did help to release more GLR from the cell. In comparison of microwave cooking, low‐temperature cooking showed relatively lower efficiency of releasing GLR in broccoli, but the broccoli cooked at 40°C could still contain 50% more contents of GLR, suggesting that the low‐temperature either by regular cooking or by microwave heating can significantly (*p* < .05) increase the GLR levels in broccoli. Besides, in general, the levels of GLR decreased when the cooking temperature increased. This is due to the increasing temperature which could help to break more cells and release more myrosinase to hydrolyze GLR into SFR.

The levels of SFR in broccoli cooked at different temperatures were also determined (Figure 2). The broccoli cooked using microwave at 60°C showed the highest SFR level at 1.50 µmol/g DW, but microwave cooking at 70°C drastically reduced the SFR level which was not significant (*p* ≥ .05) from the level detected in raw broccoli. This might be because high temperature denatured the myrosinase which results in termination of enzymatic reaction from GLR to SFR. As compared to microwave cooking, low‐temperature cooking showed the same trend of SFR levels observed in broccoli using different cooking temperature from 40 to 70°C. The broccoli cooked at 70°C showed the least levels of SFR in all treated broccoli samples.

Generally, microwave cooking can increase the SFR contents because of the hydrolysis of GLR by myrosinase only in a certain range of cooking temperature (40–60°C) heating. The same trend was also found in low‐temperature cooking.

### 3.2 Effect of microwave power levels on GLR and SFR levels in broccoli

**Figure 3:**
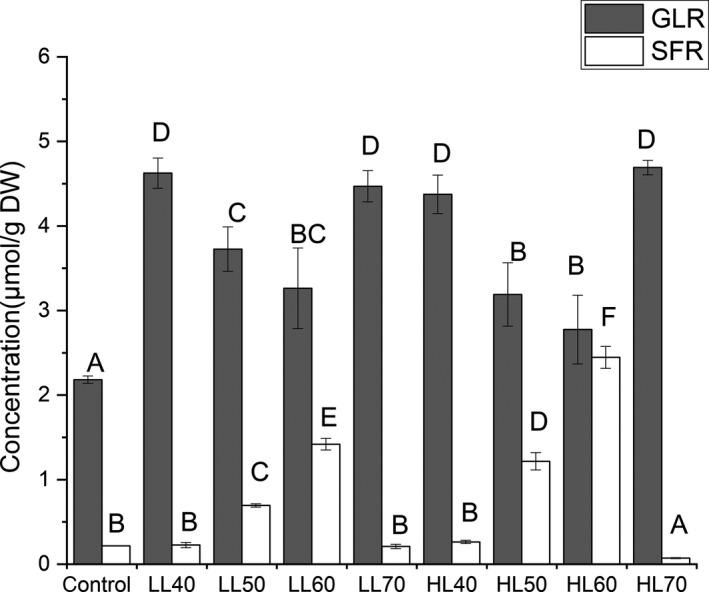
The glucoraphanin (GLR) and sulforaphane (SFR) contents (μmol/g DW) in florets of broccoli during microwaving at 40, 50, 60, and 70°C using low power level (LL) or high power level (HL). Data are reported as the mean ± *SD* (*n* = 3). Values with different letters are significantly (*p *< .05) different

Instead of considering the heating temperature is the only factor that may affect the levels of GLR and SLR in broccoli, the influence of microwave irradiation's power levels on the GLR and SLR contents were also determined. The levels of GLR and SFR in broccoli at the temperature range from 40–70°C using low and high microwave powers were shown in Figure 3.

Microwave cooking using higher power with less cooking time showed higher SFR contents than the low level with longer cooking time. For instance, the microwave using high level at 60°C showed the greatest SFR level (2.45 µmol/g DW), while the microwave using low level at 60°C showed less SFR value (1.42 µmol/g DW). However, when temperature increased to 70°C, the SFR levels in broccoli microwaved at high level were lower than that treated at low level and it was even lower than that of control. This indicates that 70°C microwave cooking may degrade the SFR of broccoli. According to Pearson's correlation analysis (Table 1), both GLR and SFR contents were positively correlated with microwave power level (*r* = .948 for GLR, *r* = .990 for SFR). In the temperature range of 50–60°C, a positive correlation was observed between GLR or SFR contents and temperature. However, these two physiochemical contents were negatively correlated with temperature when it increased to 70°C. These results indicate that microwave power level plays a dominant role in influencing GLR and SFR contents of broccoli, while temperature only positively affects their levels in a certain range.

**Table 1:** Correlation (Pearson *r* value) between glucoraphanin (GLR) and sulforaphane (SFR) and microwave conditions including temperature (*T*) and microwave power level^a^

	*T* (40–50°C)	*T* (50–60°C)	*T* (60–70°C)	Power level
GLR	−0.098	0.948**	−0.591	0.948**
SFR	0.568	0.969**	−0.959**	0.990**

^a^ Data are expressed as Pearson correlation coefficients (*r* value).

** *p* < .01, values without asterisks are not significant different at*p* < .05.

### 3.3 Effect of cooking on GLR and SFR levels in different broccoli parts

**Figure 4:**
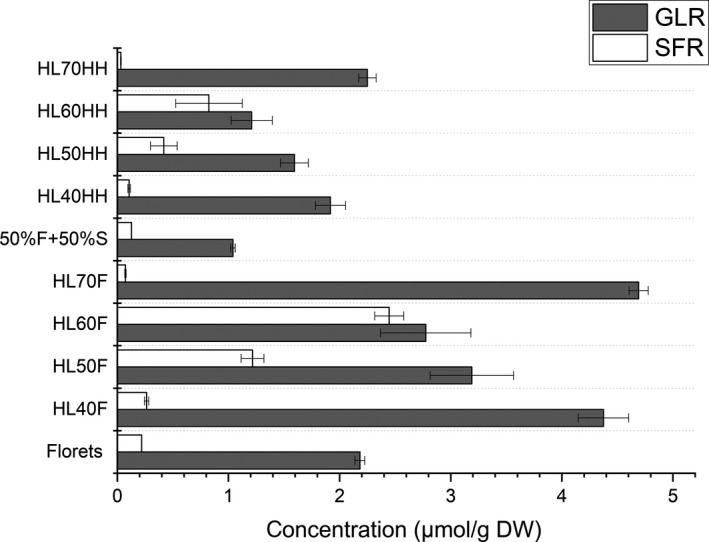
The glucoraphanin (GLR) and sulforaphane (SFR) contents in florets (F) of broccoli and in mixtures of florets and stems with 1:1 ratio (HH) during microwaving at 40, 50, 60, and 70°C using high power level (HL). Data are reported as the mean ± *SD* (*n* = 3). No treatment controls for mixtures of florets and stems and florets only are labeled as 50%F + 50%S and florets, respectively

We further analyzed the concentrations of GLR and SFR in stems and in mixture of florets and stem (1:1) during microwave treatment at high power level. GLR and SFR were hardly detected in stems (data not shown). Less than 52% of GLR was detected in the mixture of florets and stems compared to florets (Figure 4). Under microwaved at 60°C, the florets had a concentration of GLR and SFR at 2.78 and 2.45 µmol/g DW, respectively, which was significantly (*p* < .05) higher than the levels detected in mixture of florets and stems (1.21 and 0.82 µmol/g DW, respectively). Similar observations could be obtained for the broccoli microwaved at other temperatures, with higher levels of GLR and SFR in florets than those in mixture of florets and stems. These results indicate that GLR and SFR are present mostly in florets.

## 4 DISCUSSION

Growing interests in the health benefits of broccoli focus on SFR, an unstable isothiocyanate derived from GLR (Lu et al., 2018). Broccoli is often consumed after cooking, which could induce changes in these phytochemical contents. As a popular domestic cooking method with little or no water needed, microwave treatment causes a sudden collapse of cell structure due to the increase in osmotic pressure difference over vacuole membrane, resulting in the mixing of the GLS and MYR (Palermo et al., 2014). In this study, the effects of temperature and power level of microwaving on the contents of GLR and SFR were investigated as well as the distribution of these two bioactive compounds in different parts of broccoli.

Our results showed that mild heating (40–70°C) either by microwave or conventional heating could increase the GLR level as compared to raw broccoli. Such mild heating treatment possibly induces more cell lysis, releasing GSL inside the vacuoles of specific cells (Nugrahedi, Verkerk, Widianarko, & Dekker, 2015). Microwave treatment can be considered a suitable option to retain GSL. For instance, Song and Thornalley (2007) found that microwaving (900 W) did not produce significant loss of GSL in broccoli for up to 3 min duration. With longer treatment time for 19 min at 950 W, microwave heating still had no effect on the total GSL level in broccoli (Tab art et al., 2018). By contrast, Jones et al. (2010) reported a significant reduction (15%‐17%) of GLR contents in broccoli after 5 min of microwaving at 1,100 W due to leaching of GLR into cooking water. At the same microwave condition, Yuan et al. (2009) also detected loss of total GSL in broccoli, and the loss of GLR was 62%. The controversial results could be explained by different microwave conditions such as power level, treatment time, volume of water added, and sample sizes.

The effect of microwaving on SFR contents was dependent on temperature, with the maximum level of SFR achieved at 60°C and the lowest level obtained at 70°C. In agreement with this finding, Matusheski et al. (2004) found enhanced SFR formation in preheated broccoli florets to 60°C for 5 min and 10 min treatments and decreased level of SFR in 70 or 100°C heated broccoli. This indicates that mild heating could increase SFR level, possibly explained by the increased activity of MYR which can hydrolyze GLR into SFR at high temperature (up to 60°C). This can be supported by the observation of decreased concentration of GLR when temperature increased from 40 to 60°C. Moreover, SFR is not the only hydrolysis product of GLR, which can produce nitriles with the action of epithiospecifier. This enzyme could be inactivated by heating at 60–70°C for 5–10 min, while MYR was inactivated at 100°C for 5–15 min (Jones et al., 2006). Thus, the reduced activity of epithiospecifier by mild heating could decrease the production of sulforaphane nitrile and allow for more production of SFR.

Microwave could retain GLR and SFR levels of broccoli compared to conventional heating in water, although the same temperatures were applied in these two methods. Instead of heat conduction, volumetric heating is the most important property of microwave which means faster heating rates and uniform heating (Vadivambal & Jayas, 2007). Internal temperature of broccoli could increase faster during microwave cooking compared to other cooking methods such as boiling and steaming (Jones et al., 2010); thus, less time is needed to reach the same temperature for microwaving compared to low‐temperature heating method. This possibly reduces the duration of leaching these compounds into the cooking water. However, the exact mechanisms for the less degradation of GLR and SFR in broccoli during microwave treatment were still unknown and need further research.

Regarding microwave power level, our results indicated that SFR amount in broccoli was positively correlated with microwave power level. High power level could promote the production of SFR in broccoli compared to low power level. Effect of microwave power on GSL content was previously reported by López‐Berenguer, Carvajal, Moreno, and García‐Viguera (2007) who found great losses of total GSL as well as the individual GLR at 500 W compared to 1,000 W and 700 W for 5 min. They further explained that the high power set might prevent the MYR from hydrolyzing GSL, with high level retained in the broccoli. This could be related to the inactivation of MYR at high power level, which can be supported by the findings of Verkerk and Dekker (2004) who reported that great activity of MYR was retained in cabbage treated at 180 W in comparison to the complete loss of activity at 900 W. However, in the present study, the inactivation of MYR during microwave at high power set did not occur due to the temperature control. To reach the same temperature about 50–60°C, less time is needed for high microwave power level than low level. The effect of microwave time on SFR level has been reported by Wang et al. (2012) who found that microwave heating (900 W) for 0.5 and 0.75 min increased the SFR production, while 1 min treatment decreased the level from the maximum. With even longer treatment time, Galgano et al. (2007) found a 39% reduction of SFR in broccoli cooked by microwave (600 W) for 11 min. Thus, the greater contents of SFR in broccoli microwaved at high level could be related with the low chance of leaching into cooking water during shorter heating process.

Although stems are usually cut from the broccoli before cooking, the stems can be consumed by people due to the presence of antioxidant components such as great vitamin C contents (Zhang & Hamauzu, 2004). However, to the best of our knowledge, no studies have compared the GSL levels in different parts of broccoli during domestic cooking. In the present study, higher levels of both GLR and SFR were detected in florets compared to the stems during microwave cooking. In accordance with previous studies (Jia et al., 2009; Liang, Yuan, Dong, & Liu, 2006), GLR and SFR were present at higher concentrations in the floret of fresh broccoli, with the florets: stem ratio of 1:0.7 (Galgano et al., 2007).

## 5 CONCLUSIONS

This study investigated the changes in GLR and SFR levels of broccoli during microwaving and low‐temperature heating. Although mild heating (40–60°C) either by microwave or conventional heating could increase the GLR and SFR contents in broccoli, microwave‐cooked broccoli had higher levels of these two compounds compared to broccoli heated in water. The temperature could affect GLR and SFR contents in broccoli, with lower GLR concentration at higher temperature and the maximum level of SFR obtained at 60°C. The SFR contents were positively correlated with microwave power level, with higher SFR level found in broccoli microwaved at high level compared to low level. In addition, GLR and SFR are mostly present in the broccoli florets rather than in stems. The present study suggests that consuming microwave‐cooked broccoli florets at certain condition (50–60°C, high power level) could achieve high bioavailability of GLR and SFR.

## CONFLICT OF INTEREST

The authors declare that they do not have any conflict of interest.

## ETHICAL STATEMENT

This study does not involve any human or animal testing.

## Supporting information

Table S1
