# Supplementary material for: Microwave cooking increases sulforaphane level in broccoli
Source: Food Sci Nutr. 2020 Mar 5;8(4):2052–8. doi: 10.1002/fsn3.1493 (PMC7174218; doi:10.1002/fsn3.1493)
Supplement: Supplementary file 1 — Table S1 [file FSN3-8-2052-s001.docx]

**Supplementary Materials**

**Table S1**. Parameters for cooking treatments

|  | Temperature  (°C) | Time  (S) | Power level  (W) |
| --- | --- | --- | --- |
| Heating in water | 40 | 185 | NA |
|  | 50 | 230 |  |
|  | 60 | 262 |  |
|  | 70 | 290 |  |
| Microwave (HL) | 40 | 65 | 950 |
|  | 50 | 90 |  |
|  | 60 | 108 |  |
|  | 70 | 120 |  |
| Microwave (LL) | 40 | 115 | 475 |
|  | 50 | 148 |  |
|  | 60 | 178 |  |
|  | 70 | 200 |  |
